# Integrative multi-omics characterization reveals sex differences in glioblastoma

**DOI:** 10.1186/s13293-024-00601-7

**Published:** 2024-03-16

**Authors:** Byunghyun Jang, Dayoung Yoon, Ji Yoon Lee, Jiwon Kim, Jisoo Hong, Harim Koo, Jason K. Sa

**Affiliations:** 1 Department of Biomedical Informatics, Korea University College of Medicine, Seoul, South Korea; 2 Department of Biomedical Sciences, Korea University College of Medicine, Seoul, South Korea; 3 Department of Cancer Biomedical Science, Graduate School of Cancer Science and Policy, National Cancer Center, Goyang, South Korea; 4 Department of Clinical Research, Research Institute and Hospital, National Cancer Center, Goyang, South Korea

**Keywords:** Multi-omics, Glioblastoma, Sex-difference, Proteomics

## Abstract

**Background:**

Glioblastoma (GBM) is the most common and lethal primary brain tumor in adults, with limited treatment modalities and poor prognosis. Recent studies have highlighted the importance of considering sex differences in cancer incidence, prognosis, molecular disparities, and treatment outcomes across various tumor types, including colorectal adenocarcinoma, lung adenocarcinoma, and GBM.

**Methods:**

We performed comprehensive analyses of large-scale multi-omics data (genomic, transcriptomic, and proteomic data) from TCGA, GLASS, and CPTAC to investigate the genetic and molecular determinants that contribute to the unique clinical properties of male and female GBM patients.

**Results:**

Our results revealed several key differences, including enrichments of MGMT promoter methylation, which correlated with increased overall and post-recurrence survival and improved response to chemotherapy in female patients. Moreover, female GBM exhibited a higher degree of genomic instability, including aneuploidy and tumor mutational burden. Integrative proteomic and phosphor-proteomic characterization uncovered sex-specific protein abundance and phosphorylation activities, including EGFR activation in males and SPP1 hyperphosphorylation in female patients. Lastly, the identified sex-specific biomarkers demonstrated prognostic significance, suggesting their potential as therapeutic targets.

**Conclusions:**

Collectively, our study provides unprecedented insights into the fundamental modulators of tumor progression and clinical outcomes between male and female GBM patients and facilitates sex-specific treatment interventions.

**Highlights**

- Female GBM patients were characterized by increased MGMT promoter methylation and favorable clinical outcomes compared to male patients.
- Female GBMs exhibited higher levels of genomic instability, including aneuploidy and TMB.
- Each sex-specific GBM is characterized by unique pathway dysregulations and molecular subtypes.
- EGFR activation is prevalent in male patients, while female patients are marked by SPP1 hyperphosphorylation.

**Supplementary Information:**

The online version contains supplementary material available at 10.1186/s13293-024-00601-7.

## Plain language summary

Glioblastoma (GBM) is the most common and lethal primary brain tumor in adults with limited treatment modalities and dismal prognosis. A thorough understanding of sex differences could facilitate personalized therapeutic strategies in GBM. In this study, we conducted a comprehensive multi-omics analysis from TCGA, CPTAC, and GLASS studies, revealing distinct molecular and clinical disparities between male and female GBM patients. We discovered that female GBM patients exhibited enrichments of MGMT promoter methylation and high genomic instability, including aneuploidy and TMB. While male GBMs were characterized by activation of EGFR protein and phosphorylation activities, female GBM patients demonstrated hyperphosphorylation of SPP1. Notably, these proteins demonstrated prognostic significance, highlighting their potential as therapeutic targets. Our findings provide unprecedented insights into the fundamental modulators of tumor progression and clinical outcomes in male and female GBM patients, offering valuable opportunities for sex-specific treatment interventions.

**Supplementary Information:**

The online version contains supplementary material available at 10.1186/s13293-024-00601-7.

## Background

Gliomas are tumors that arise from the supportive tissue of the brain and are graded based on their histopathological characteristics [1]. Among them, Glioblastoma (GBM) is the most common and lethal primary brain tumor in adults with a median survival of less than 15–18 months and a 5-year survival rate of only 5% [2, 3], despite aggressive treatment modalities, including surgical resection, chemotherapy and radiotherapy [4, 5]. GBMs can be subcategorized into distinct molecular subtypes based on their transcriptional cellular states and accompanying unique genomic alterations [6–8]. However, despite continuous efforts in treatment innovations, GBM still remains therapeutically unresolved due to its complex genomic architecture [9–11].

Recent studies have systematically examined the clinical impacts of sex differences on cancer incidence, prognosis, and treatment outcomes across a wide spectrum of different tumor types, including colorectal adenocarcinoma, melanoma, lung adenocarcinoma, and GBM [12–15]. GBM is characterized by the predominance in male populations with a male-to-female ratio of approximately 1.5 to 1 [2, 3], suggesting that sex-hormonal differences could potentially affect tumor propagation and progression [16, 17]. Furthermore, several experimental studies have made efforts to evaluate the involvement of estrogen receptors and testosterone in GBM malignancy [18, 19] and revealed that female GBM patients generally respond better to standard treatments, including chemotherapy [20, 21] and radiotherapy [22]. Moreover, recent studies have identified the functional impacts of key driver alterations, including *RB* activation and *ADCY8* mutation in the sexual dimorphism of GBM, and discovered that diffuse gliomas are characterized by sex-biased mutation clonality [23]. However, due to the limited amount of information that can be acquired from genomics alone, underlying molecular mechanisms that drive the unique malignant transformation of GBM between the two sexes still remain elusive. Therefore, integrative multi-omics analyses on the sex differences could provide unprecedented insights into the fundamental modulators of tumor progression and clinical outcomes in GBM and facilitate sex-specific treatment interventions.

In the present study, we leveraged large-scale multi-omics data, including genomics, transcriptomics, proteomics, and phospho-proteomics from TCGA, GLASS, and CPTAC datasets to identify key genetic determinants that constitute unique molecular and clinical properties between male and female GBM patients. Our results could potentially open up new therapeutic opportunities for considering sex differences in the treatment of GBM.

## Materials and methods

### GBM data acquisition

The following three independent datasets were used in this study: The Cancer Genome Atlas (TCGA), Clinical Proteomic Tumor Analysis Consortium (CPTAC), and Glioma Longitudinal AnalySiS (GLASS). TCGA dataset was acquired from the cBioPortal and UCSC Xena database, and the CPTAC dataset was downloaded from the cBioPortal and GDC database. The GLASS cohort was obtained from the GLASS consortium publications [24].

### Somatic mutation analysis

In order to analyze somatic mutations in glioblastoma, we downloaded the maf file containing data from 245 male and 143 female patients in TCGA GBM. To identify significant somatic mutations in each sex, we utilized MutSig2CV. This analysis tool identifies genes that are mutated more frequently than expected by chance, taking into account background mutational processes and other covariates. Genes were considered significantly mutated if they had a false discovery rate of q < 0.1, corrected for multiple hypothesis testing.

### Copy number alteration

To analyze copy number alterations (CNA) in glioblastoma, we downloaded the seg file containing data from 348 male and 225 female patients in TCGA GBM. To identify CNAs at the chromosome arm level, we utilized GISTIC2.0. This tool identifies regions of the genome that are significantly amplified or deleted across various samples. Each aberration is assigned a G-score that considers the amplitude and frequency of its occurrence across samples. False Discovery Rate (FDR) q-values are then calculated for the aberrant regions, and regions with q-values below a user-defined threshold are considered significant. Amplification and deletion were defined as having a log2 ratio of ≥ 1 and ≤ − 1. The q-value ≤ 0.25 is determined by the significance threshold.

### Gene set enrichment analysis (GSEA)

We used GSEA to identify significantly enriched genomic signatures in each sexual group. GSEAs were performed using the GSEA Java application, which was downloaded from the Broad Institute website (https://www.gsea-msigdb.org/gsea/index.jsp). We searched for gene sets in Human MSigDB, which includes Reactome, Gene Ontology (GO), Human Phenotype Ontology (HPO), and WikiPathways. We selected two gene sets that had important functions in the significant gene sets identified in male and female patients.

### Single-sample gene set enrichment analysis (ssGSEA)

ssGSEA is an adaptation of Gene Set Enrichment Analysis that generates an enrichment score for a given gene set in an individual sample. Each score represents the extent to which the genes in a given set are either up- or downregulated in a single sample. We performed ssGSEA by generating an input file that consisted of normalized gene expression data across samples, and an enrichment score was computed based on a list of MSigDB gene sets, including Reactome, GO, HPO, and WikiPathways. A bigger dot plot indicates a significantly enriched gene set in males and females.

### Tumor microenvironment analysis

xCell was used to estimate the presence of different immune cells and brain normal cells in the tumor and to calculate an immune score. xCell scores in 18 immune cell types (CD4 Tcm, CD4 Tem, CD8 naive T cells, Tregs, Th1 cells, Th2 cells, B cells, memory B cells, monocytes, macrophages, macrophages M1, macrophages M2, dendritic cells, cDC, pDC, neutrophils, Mast cells, NKT) and 2 brain cell types (Astrocyte, neurons) are analyzed. The immune score is a sum of all of the immune cell scores. Strom score is a sum of all of the other cell scores. The microenvironment score is the immune score + stroma score.

### DAVID gene ontology enrichment analysis

The 798 and 829 genes, which were uniquely enriched in male and female patients, were identified using gene symbols were uploaded to DAVID (http://david.abcc.ncifcrf.gov/) and the enrichment analyses of Kyoto Encyclopedia of Gene and Genome (KEGG) and Gene ontology (GO) terms including biological process, molecular function, and cellular component were performed by using the functional clustering annotation tools. The default options with high classification stringency were used, and finally, cluster names were extracted from the most biologically relevant KEGG and GO term assigned to that cluster.

### Statistical analysis

T-test, Wilcoxon rank-sum test, Pearson correlation coefficient test, and Fisher’s exact test were used to two categorical variables analyses. Survival analyses were performed using the Kaplan–Meier method and the Cox proportional hazards regression method. These analyses considered patients who survived the last known follow-up to be censored. Hazard ratios (HR) and their 95% confidence intervals were calculated. All statistical analyses were conducted using R (version 4.1.2) software.

## Results

### Sex difference in GBM reveals favorable clinical outcomes in females

**Fig. 1:**
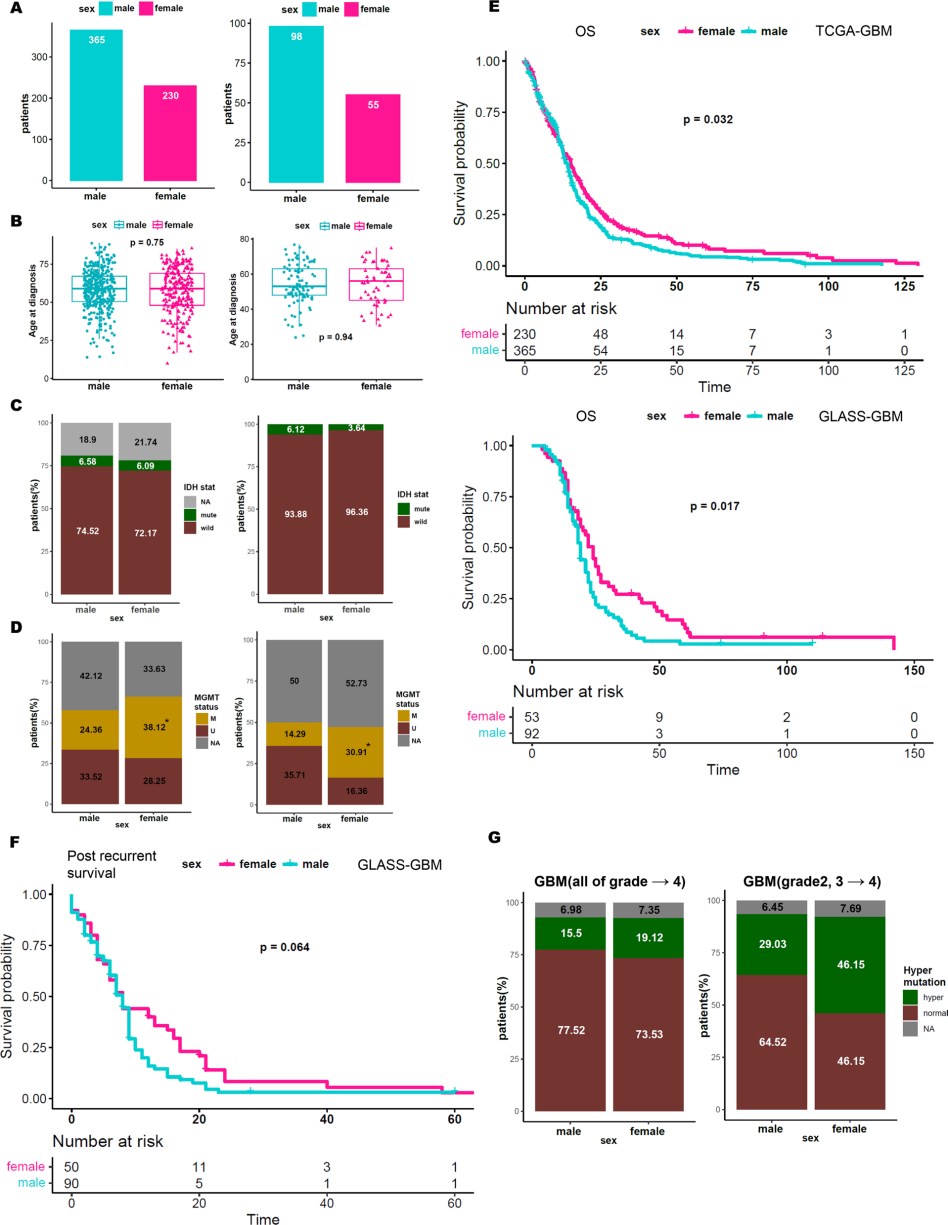
Molecular and clinical feature differences in GBM by sex. **A–D** Patient counts **(A)**, patient age **(B)**, IDH mutation ratio **(C)**, and MGMT status **(D)** were obtained from The Cancer Genome Atlas (TCGA) (left) and The Glioma Longitudinal Analysis (GLASS) (right). M represents MGMT promoter methylation, and U represents MGMT promoter unmethylation. **E** Overall survival was analyzed using Kaplan–Meier in a dataset of male and female patients from TCGA and GLASS. **F** Post-recurrent survival was analyzed using Kaplan–Meier in a dataset of male and female patients from GLASS. **G** Hypermutation (more than 10 mutations per Mb) ratio was obtained in all GLASS recurrent GBM patients and deteriorated patients of GLASS. *p < 0.05

A total of 740 patients that were diagnosed with GBM, including grade 4 astrocytoma (IDH-mutant) and glioblastoma (IDH-wildtype), from TCGA and GLASS cohorts were analyzed. Among them, the overall male-to-female ratio was 1.59, and 1.78 in TCGA and GLASS studies, respectively (Fig. 1A). While the patient age at diagnosis or *IDH1* mutational status did not show much difference between males and females in both cohorts (Fig. 1B, C), female patients demonstrated significant enrichments of MGMT promoter methylation (Fig. 1D). Moreover, we found that MGMT promoter methylation was more prevalent in female GBMs in the Chinese Glioma Genomic Atlas as well (Additional file 1: Figure S1). When we specifically focused only on the IDH-wildtype GBMs, the results remained consistent (Additional file 1: Figure S2). MGMT promoter methylation was directly associated with increased overall survival of female GBM patients in both TCGA (p-value = 0.032) and GLASS cohorts (p-value = 0.017) (Fig. 1E). On the contrary, low-grade gliomas (LGG) or diffuse gliomas did not show significant differences in survival between males and females (Additional file 1: Figure S3). Interestingly, when we analyzed the disease progression rate of longitudinal GBM patients in the GLASS cohort, we discovered that female patients demonstrated a longer post-recurrence survival rate (Fig. 1F), while there was no difference in disease-free survival duration after the initial treatment (Additional file 1: Figure S4). Furthermore, we discovered that female patients, characterized by hypermethylation of *MGMT*, were more susceptible to acquiring a hypermutator phenotype at recurrence compared to male GBM patients (Fig. 1G), which was consistent with previous reports where MGMT silencing leads to impairment of TMZ-induced mutagenesis [25]. Collectively, our results underscore that sex disparity attributes to the distinct clinical outcomes of GBM patients.

### Molecular disparity between males and females in GBM

**Fig. 2:**
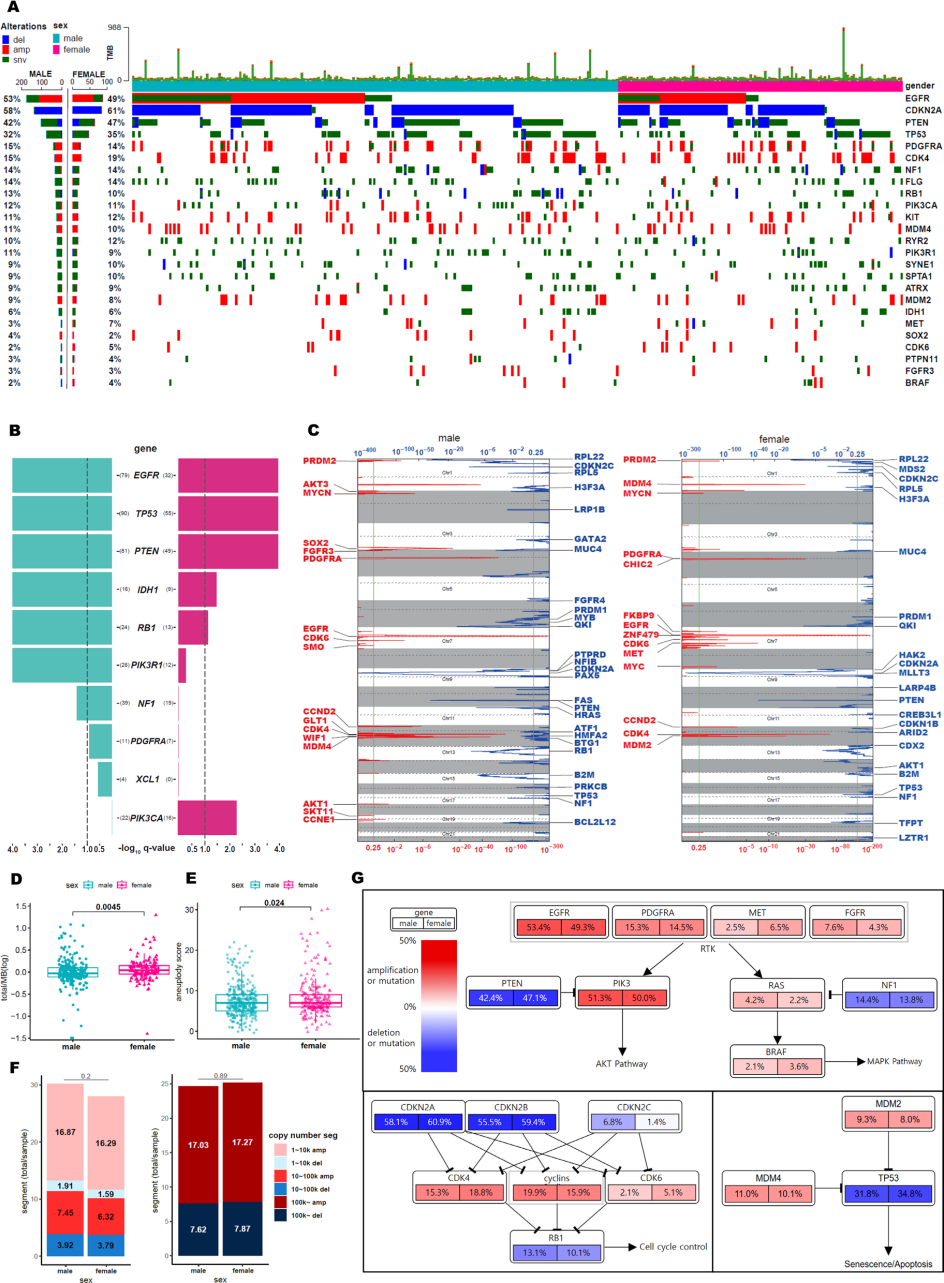
Sex differences in DNA mutation and copy number alterations. **A** Gene mutation and copy number landscape of GBM were analyzed based on sex. Genes known to be important in GBM were considered. **B** MutsigCV q-value data was analyzed. Turquoise represents significance in males, pink represents significance in females. The line represents the significance cutoff at a q-value of 0.1. **C** GISTIC heatmap was used to show the genomic copy number profiles from the GBM cohort in TCGA. The gain (red) and loss (blue) of each peak were shown. The x-axis represents the significance of the q-value. The top is the q-value of loss, the bottom is the q-value of gain. The green line represents the significance cutoff at a q-value of 0.25. **D** Tumor mutation burden (TMB) value was analyzed in males and females, and the p-value was calculated using the Wilcoxon test. **E** Aneuploidy score was analyzed in males and females, and the p-value was calculated using a t-test. **F** Copy number segment count value was analyzed in males and females. 10 ~ 100 k amplification (red), 100 k ~ amplification (dark red), 1 ~ 10 k deletion (sky), 10 ~ 100 k deletion (blue), 100 k ~ deletion (dark blue). **G** Gene mutation and significant copy number changes for the RTK/RAS/PI(3)K, p53, and cell cycle signaling pathways were shown. Red represents activating alteration; blue represents inactivating alteration. Deepening shade shows frequency. The left indicates male, and the right indicates female

Previous large-scale genomic studies have collectively identified essential genomic alterations that were frequently dysregulated in GBM, including somatic mutations and/or copy number alterations in *EGFR, PTEN, TP53, CDKN2A,* etc. [11, 26–28]. These molecular aberrations constituted the unique and complex hierarchy of GBM genomic architecture. To determine if sex differences affect the prevalent abnormality in the molecular structure of GBM, we investigated the genomic profiles, including somatic mutations and copy number variations, of TCGA GBM patients (n = 387) (Fig. 2A). While the overall genomic alteration frequency did not show much difference between males and females as both groups demonstrated recurrent genomic aberrations in the *EGFR, CDKN2A, PTEN,* and *TP53* genes*,* we discovered that several gain-of-function mutations were considerably sex-specific (Fig. 2B). For example, mutations in *PIK3R1* and *NF1* were highly enriched in male GBMs, whereas *PIK3CA* mutations were predominantly found in female patients, suggesting that activation of the PI3K-AKT-mTOR pathway is modulated through different paths between the two sexes. Notably, *PIK3CA* mutations have been previously identified as a direct drug target [29], while *PIK3R1* mutations conferred increased sensitivities to MEK inhibitors [30, 31]. As such, the clinical utility of these compounds could further guide sex-specific treatment opportunities. Furthermore, when we interrogated the chromosomal-level genomic ablation events, we found that genomic amplifications of *AKT1* (chr17) and *LRP1B* (chr2) deletions were more frequently observed in male patients (Fig. 2C). Conversely, female patients were marked by focal amplification of *MYC* (chr8) and genomic deletion of *LZTR1* (chr21), highlighting that female tumors are largely sustained by cell cycle progression and proliferation.

We assessed overall genomic instability between male and female GBM patients and discovered that females harbored a higher degree of aneuploidy fraction as well as tumor mutational burden (TMB) (Fig. 2D, E). Furthermore, the number of small copy number variation segments (< 100 k) was highly evident in male patients whereas there was no difference in the degree of large copy number variation segments (> 100 K) (Fig. 2F). As several canonical oncogenic pathways are frequently dysregulated in GBM, including RTK-RAS, PI3K, p53, and cell cycle signaling pathways [11, 32], we further assessed whether there was any major sex disparity. Unfortunately, we found no evidence of such dimorphism between male and female tumors (Fig. 2G).

### Identification of sex-specific cellular signaling pathways and transcriptional classification in GBM

**Fig. 3:**
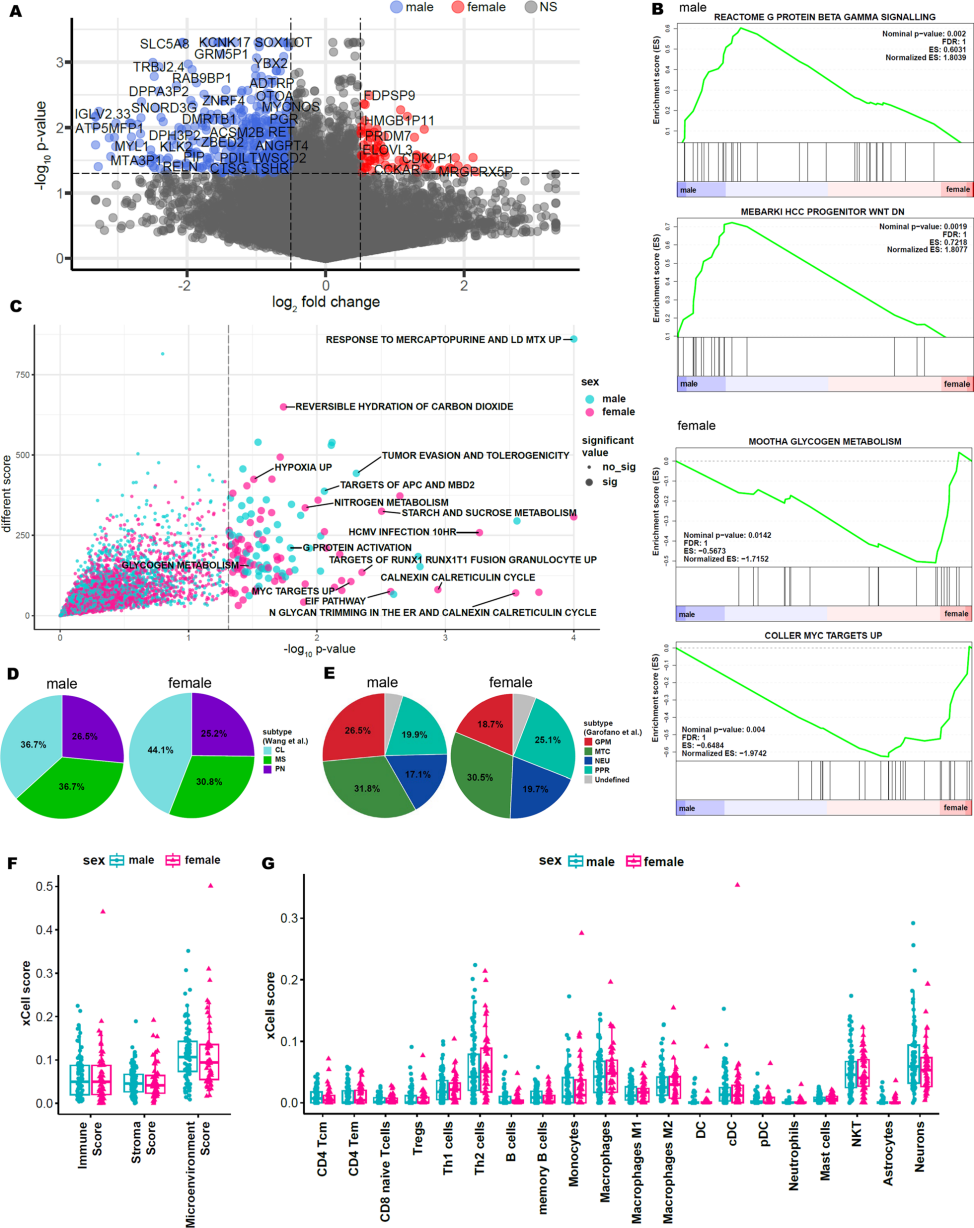
Sex-specific transcriptome difference and pathway enrichment. **A** Analysis of differential gene expression in different sexes. The cutoff for log2fold change is 0.4, and the cutoff for p-value is 0.05. **B** Gene set enrichment analysis in different sexes. The gene set database used is the Human MSigDB, which includes Reactome, GO, and Wikipathways. **C** Single-sample gene set enrichment analysis (ssGSEA) of males and females. The significant gene sets are identified based on a p-value < 0.05. **D** The percentage of GBM transcriptional subtypes based on TCGA in patients (Wang et al.). Green represents MS (mesenchymal), purple represents PN (proneural), and cyan represents CL (classical). **E** The percentage of GBM pathway-based subtypes in patients (Garofano et al.). Red represents GPM (glycolytic/plurimetabolic), green represents MTC (mitochondrial), blue represents NEU (neuronal), and cyan represents PPR (proliferative/progenitor). **F, G** Immune cell type enrichment analysis of RNA expression data using the xCell tool. **F** Scores for three cell types. **G** Scores for immune cells that are important in GBM

To identify underlying transcriptional cellular signatures or pathways that constitute the unique biology of male and female GBMs, we performed genome-wide differentially expressed gene analysis. Among a list of transcriptomes that were highly enriched, we selected candidate genes that were previously annotated in the OncoKB knowledge database as proto-oncogenes, including *PGR*, *TSHR*, *RET*, *KLK2*, and *RELN*, which were significantly expressed in male populations (Fig. 3A). To explore the functional relevance of the identified transcriptomes, we leveraged over 6,000 pathway genesets from The Molecular Signatures Database (MSigDB) and quantified each signature’s pathway activities. Notably, male GBMs were characterized by activation of the G-protein and WNT signaling pathways, while female patients demonstrated enrichments of pathways that were associated with metabolism activity and cell cycle kinetics via MYC targets (Fig. 3B, C).

Previous studies have identified transcriptome-based subtypes with distinct clinical outcomes and responses to therapies. Notably, among these subtypes, the mesenchymal subtype has been consistently associated with poor survival outcomes. [6–8, 33]. Such methods have emerged as an important concept in indicating patient prognosis as well as pharmacological vulnerability. Therefore, to determine the composition of transcriptome-based subtypes between male and female GBMs, we measured the gene expression profiles of each core signature activity [8]. Interestingly, while male GBMs were characterized by enrichments of the mesenchymal subtype (high treatment resistance), female patients were mainly composed of the classical type (Fig. 3D). These results were further corroborated through a pathway-based classification system, where glycolytic/plurimetabolic pathways were considerably more enriched in the male populations compared to females and female patients showed a higher frequency of proliferative/progenitor subtypes [6] (Fig. 3E). However, as the prevalence of expression-based subtypes is largely affected by sampling location, including the prevalence of mesenchymal subtypes in the peri-necrotic tumor areas, the results should be interpreted cautiously. We also determined the effects of sex differences in tumor microenvironment composition through immune/stromal deconvolution analysis. Although male GBMs demonstrated considerable levels of microenvironment scores, including CD4 T cells, memory B cells, and NK cells, they weren’t statistically significant (Fig. 3F, G).

### Differential protein and phosphor-protein abundance reveals enrichments of EGFR in males and SPP1 in females

**Fig. 4:**
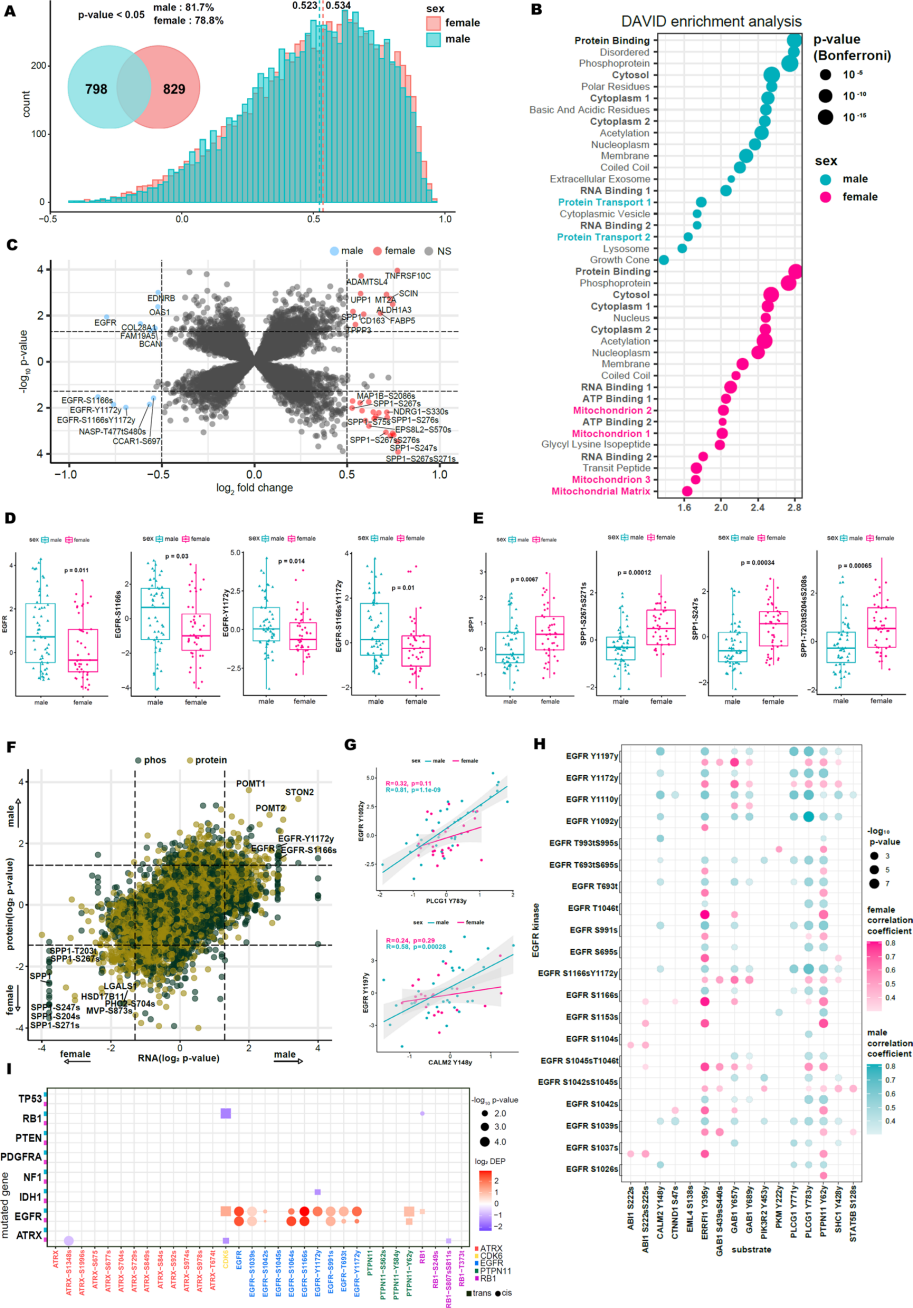
Protein enrichments and phosphorylation activity differences in males and females. **A** Histogram of Pearson correlation values of protein and RNA expression. Venn diagram shows a unique gene list that significantly correlated (p-value < 0.05, correlation > median) in each group. **B** DAVID Gene Ontology enrichment analysis for the significantly correlated unique gene list in males (top) and females (bottom). The data show the top 20 gene sets by p-value. The x-axis is log10 (annotated gene count). **C** Analysis of differentially expressed proteins and phosphorylated proteins in males and females with GBM. The cutoff for log2 fold change is 0.5 and the cutoff for p-value is 0.05. **D** Abundance of EGFR and phosphorylated EGFR proteins in males and females. **E** Abundance of SPP1 and phosphorylated SPP1 proteins in males and females. **F** Comparison of significant p-values for protein and phosphorylated protein to RNA in females and males. **G** EGFR Y1092-PLCG1 Y783 and EGFR Y119-CALM2 Y148 correlated expression. **H** 20 EGFR kinase and 16 substrates significant correlation. Turquoise is significant in males, pink is significant in females. **I** Cis–trans effects of mutated core genes (y-axis) in GBM on protein and phosphorylated protein levels (x-axis)

The central dogma of molecular biology elucidates the flow of information from DNA to mRNA and to proteins. When combined with genomics and transcriptomics, proteomics delivers a deeper understanding of cancer biology that has gone largely unnoticed by genomics and/or transcriptomics studies alone [27, 34, 35]. To uncover the underlying molecular mechanisms that encapsulate the sex-oriented disparity in GBM, we adopted the integration of genomics and transcriptomics with deep proteomics characterization using the dataset from the Clinical Proteomic Tumor Analysis Consortium (CPTAC). To explore the potential post-transcriptional regulation, we first examined the proportion of genes that demonstrated concordance between mRNA to protein expression levels (Fig. 4A). Notably, most of the genes demonstrated positive mRNA-protein correlations and the median correlation for the male population was at 0.523, while the female was at 0.534. Females maintained a higher degree of concordance compared to males. Among highly correlated genes, we identified 798 and 829 genes that were uniquely enriched in male and female patients, respectively. We investigated the functional relevance of the uniquely enriched genes through Gene Ontology (GO) analysis (Fig. 4B). Interestingly, despite extracting completely different genesets, both male and female groups demonstrated activation of pathways that were associated with cytosol, cytoplasm, RNA binding, and protein binding, suggesting that while the conserved genes were different, their relative functions remained the same. On the contrary, the protein transport-associated pathway was highly enriched only in the male populations whereas mitochondrion activity was prevalent in female patients.

Next, we performed differentially expressed protein and phospho-protein analysis and uncovered OAS1 and TNFRSF10C proteins, and NASP and EPS8L2 phosphor-proteins that were increased in males and females, respectively (Fig. 4C). Remarkably, we discovered that the global expression of EGFR and its phosphorylation activities at various residues, including serine (S) and tyrosine (Y) were significantly enriched in male GBMs, indicating activation of the EGFR signaling pathway, while female GBM patients were characterized by hyperphosphorylation of SPP1 (Fig. 4D, E). Increased activities of both EGFR and SPP1 were concurrently observed in mRNA expression as well (Fig. 4F). To further determine the impact of EGFR kinase activity, we performed a kinase-substrate interaction by curating EGFR kinase abundance from proteomics data and its substrate abundance from phosphor-proteomics results (Fig. 4G, H). Among several known substrates, CALM2 phosphosites at Y148, and PLCG1 phosphosites at Y773 and Y771, demonstrated the most robust correlations with EGFR kinase activity in male GBM patients. In addition to EGFR, males also showed a high abundance of COL28A1 and EDNRB protein expressions as well as phosphorylation of NASP and CCAR1. On the contrary, female patients were marked by activation of SPP1. As previous studies have postulated that SPP1 or osteopontin mediates infiltration of tumor-associated macrophages that promote the pro-tumorigenic potential of adjacent glioma stem cells (GSCs), we speculate that microenvironment interactions shape female GBM progression. SPP1 binds to various receptors, including CD44 which has been recognized as an essential cell surface marker of cancer stem cells, driving treatment resistance and poor prognosis in various tumors [36–40]. The OPN-CD44 interaction activates the PI3K/Akt/mTOR pathway, fostering a highly aggressive stem-cell-like phenotype in GSCs, and enhancing sphere-growing capacity and tumorigenicity. Cancer-derived SPP1 is linked to MDSC (myeloid-derived suppressor cells) immunosuppression by regulating arginase 1, NOS2, VEGF, and IL-6. Moreover, SPP1-related signals have been speculated as potential therapeutic targets for immunotherapies as SPP1 stimulation enhances PD-L1 expression in macrophages [41, 42].

Lastly, we investigated the functional impact of genetic alterations on global and phosphor-proteins abundance, both *cis-*acting (cognate gene product) and *trans*-acting (other gene products) between male and female patients (Fig. 4I). We discovered strong effects in *cis* and *trans* for *ATRX* only in the female patients, while male patients demonstrated dominant effects of *RB1* at the *trans-*acting level. Surprisingly, while both sexes exhibited robust *cis* effects of *EGFR,* its phosphorylation residues differed significantly. For example, Y1172, T693, and Y1172 phosphorylation sites, which are associated with essential biological programs such as enzymatic activity, cell growth, and cell cycle regulation were highly enriched in the males, whereas female GBMs showed activation of S1064 residue, which functional role remains less well-known. Collectively, our results provide evidence of alternative pathway activities that constitute unique biological properties of GBM between male and female patients.

### Sex-specific protein prognostic markers in GBM patients

**Fig. 5:**
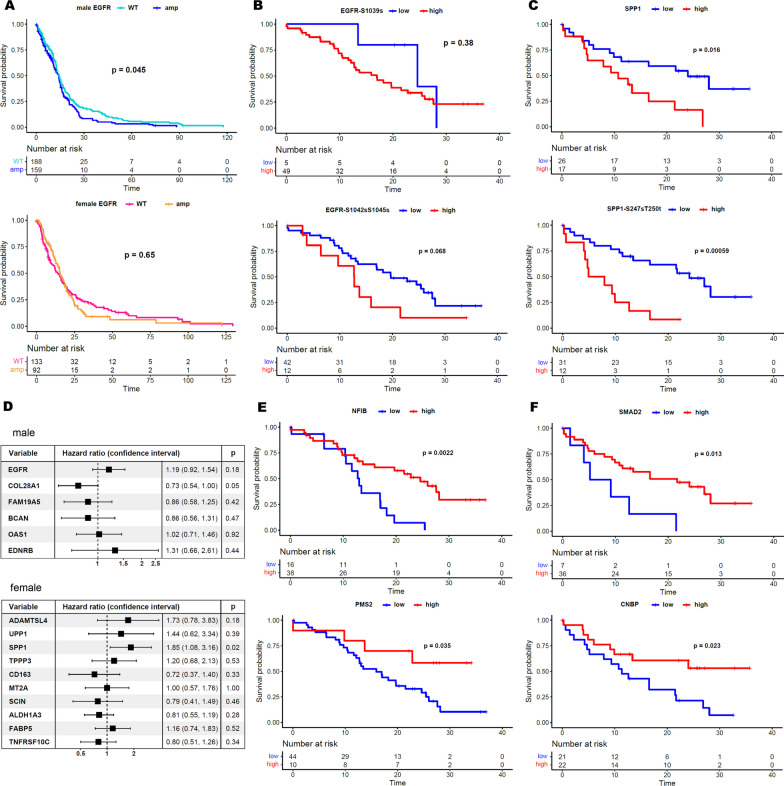
Analysis of proteins that mediate prognostic effects in each sex. **A** Overall survival effect of EGFR amplification in males (top) and females (bottom). **B** Overall survival effect of EGFR phosphorylated protein abundance in males. High and low group cutoffs are calculated by maxstat. **C** Overall survival effect of SPP1 protein and phosphorylated protein abundance in females. **D** Cox regression survival analysis on significant high-expression proteins in males and females. **E** Overall survival effect of NFIB and PMS2 proteins (significant expression and in the OncoKB gene list) abundance in males. **F** Overall survival effect of SMAD2 and CNBP proteins (significant expression and in the OncoKB gene list) abundance in females

To determine whether sex-specific biomarkers present prognostic impacts, we compared the differences in survival outcomes in male and female GBM patients. As we previously identified activation of EGFR to be the main driver of malignancy in male populations, we checked its prognostic pertinence. While *EGFR* amplification exhibited significantly worse survival outcomes in male patients, it did not present any statistical difference in female or all GBM patients (Fig. 5A). We next examined the protein and phosphor-protein abundance of both EGFR and SPP1, which we identified to drive sex-specific progression, in male and female patients, respectively. Although statistically not significant, we discovered that male patients with increased phosphorylation activity of EGFR showed worse clinical outcomes (Fig. 5B). On the contrary, high protein abundance and hyperphosphorylation of SPP1 at various residues significantly conferred worse survival probability for female GBM patients (Fig. 5C). We also identified additional proteins associated with sex-specific prognosis (Fig. 5D). Notably, the high protein abundance of COL28A1 demonstrated a favorable influence on male patients’ survival. Next, we leveraged the oncoKB knowledge database to annotate each protein’s clinical relevance and found that both NFIB (p-value = 0.0022) and PMS2 (p-value = 0.036) were associated with increased survival probabilities in males (Fig. 5E), while SMAD2 (p-value = 0.013) and CNBP (p-value = 0.023) were significantly enriched in female patients with favorable clinical outcomes (Fig. 5F). Together, these results suggest that stratification of GBM based on sex-specific approaches could provide a new innovative treatment for GBM patients.

## Discussion

Recent studies have demonstrated the profound effects of sex differences in disease progression and treatment response with high clinical applicability [12–14]. In GBM, the standard treatment has been more effective in female patients, although its underlying mechanism remains obscure [21, 43]. Therefore, new strategic treatments based on sex-specific features could facilitate personalized treatment [6, 8, 44]. Furthermore, previous studies have confirmed that GBM metabolizes glutamine using sex-specific metabolic pathways [45]. Despite continuous efforts to identify significant molecular differences between male and female GBM patients, there has yet to be a prominent implementation of sex-specific treatment opportunities in clinical practice [3].

Proteomics provides a comprehensive understanding of the complex cellular structures and functions involved in the disease [46, 47]. Although sex-specific genomic and transcriptomic characterization has been thoroughly investigated in GBM [11, 43, 48], the functional relevance of protein and phosphorylated proteins remains elusive. In this study, we utilized large-scale multi-omics data from TCGA, GLASS, and CPTAC studies to identify sex-specific features that distinguish unique genetic profiles between male and female GBM patients. As a result, we identified a significant difference in terms of overall and post-recurrence survival, where female patients demonstrated favorable outcomes, potentially due to enrichment of MGMT promoter methylation. The MGMT gene is involved in repairing DNA damage caused by alkylating agents like temozolomide. Patients with MGMT-positive tumors have an intact MGMT repair mechanism, allowing them to efficiently repair the DNA damage induced by TMZ [49]. Our comparison of the genomic and transcriptomic profiles of male and female GBM patients revealed several notable differences. Specifically, female GBMs displayed prevalent genomic instability and activation of cell cycle activities, while male GBM patients were characterized by enrichments of G protein-associated pathways and mesenchymal-like characteristics. However, when we compared these profiles at the core oncogenic pathway levels, including RTK-RAS, PI3K, Cell Cycle, and p53, we did not identify any significant differences.

When combined with proteomics and phosphor-proteomics, we identified robust sex-oriented differences. Specifically, we observed an increased abundance of the SPP1 protein and its phosphorylation activities in female GBM patients, which aligns with the previous studies on its functional roles in GBM malignancy [38, 50]. Furthermore, our findings were consistent with the importance of the integrin pathway and hypoxia in females [40, 43]. SPP1 is an extracellular matrix protein expressed in numerous tissues and has been linked to the pathogenesis of malignant tumors, including GBM [36, 37, 39]. Notably, SPP1 can enhance cellular invasion, promote stem cell–like characteristics, and increase radiation resistance [51, 52]. These effects are mediated by the PI3K/AKT signaling, ERK1/ERK2 pathway, and NF-κB signaling [53, 54]. Additionally, SPP1 contributes to immunosuppression in MDSCs by regulating NOS2, VEGF, and IL-6. Stimulation of SPP1 results in an increased expression of PD-L1 in macrophages and presents potential targets for immunotherapies [42].

In contrast to our findings in females, male GBM patients demonstrated the predominance of EGFR signaling pathways, particularly through hyperphosphorylation at various residues. EGFR signaling is known to induce DNA synthesis and cellular proliferation via the activation of the MAPK pathway, PI3K signaling, and STAT transcription factors [55, 56]. Furthermore, EGFR alterations have been widely reported across various tumor types, including lung, breast, gastrointestinal tract, and GBM, and have been associated with  increased tumorigenesis [57–59]. However, as several previous clinical trials involving EGFR-mediated therapy have shown disappointing results due to various components, such as intra-tumoral heterogeneity and alternative mechanisms of action [9, 27, 60–62], future EGFR-targeted trials require a cautionary approach. Lastly, we confirmed the prognostic effects of EGFR and SPP1 on patient survival. Interestingly, *EGFR* amplification and high phosphorylation activity only conferred a survival disadvantage in male GBMs, while hyperphosphorylation of SPP1 promoted worse survival only in female patients.

### Perspectives and significance

Our multi-omics study proposes a significant role of sex disparity in the molecular profiles and clinical outcomes of GBM. These findings highlight the need for further mechanistic investigations to understand the underlying molecular biology that dictates the diverse characteristics of GBM in male and female patients. The sex-specific multi-omics determinants identified in this study could potentially inform innovative treatment strategies for GBM patients.

### Supplementary Information

**Additional file 1: Figure S1.** MGMT status between male and female GBM patients from Chinese Glioma Genomic Atlas (CGGA). M represents MGMT promoter methylation, and U represents MGMT promoter unmethylation. **Figure S2.** MGMT status between male and female IDH-wildtype GBM patients. M represents MGMT promoter methylation, and U represents MGMT promoter unmethylation. **Figure S3.** Kaplan − Meier analysis of overall survival in a dataset of LGG and glioma patients from TCGA. **Figure S4.** Kaplan − Meier analysis of progression-free survival in a dataset of male and female patients from GLASS.

## Data Availability

We leveraged publicly available data (TCGA, CPTAC, GLASS) from corresponding sources described in the Methods section.
