# Supplementary material for: Integrative multi-omics characterization reveals sex differences in glioblastoma
Source: Biol Sex Differ. 2024 Mar 16;15:23. doi: 10.1186/s13293-024-00601-7 (PMC10943869; doi:10.1186/s13293-024-00601-7)
Supplement: Supplementary file 1 — Additional file 1: Figure S1. MGMT status between male and female GBM patients from Chinese Glioma Genomic Atlas (CGGA). M represents MGMT promoter methylation, and U represents MGMT promoter unmethylation. Figure S2. MGMT status between male and female IDH-wildtype GBM patients. M represents MGMT promoter methylation, and U represents MGMT promoter unmethylation. Figure S3. Kaplan − Meier analysis of overall survival in a dataset of LGG and glioma patients from TCGA. Figure S4. Kaplan − Meier analysis of progression-free survival in a dataset of male and female patients from GLASS. [file 13293_2024_601_MOESM1_ESM.pdf]

S1

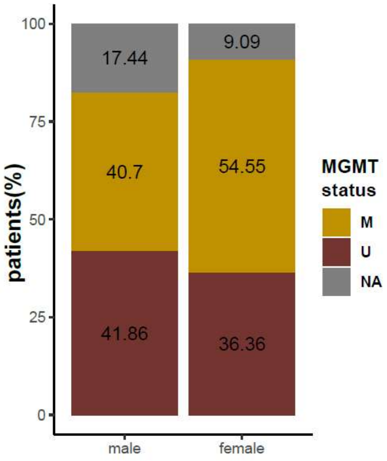

Figure S1. MGMT status between male and female GBM patients from Chinese Glioma Genomic Atlas (CGGA). M represents MGMT promoter methylation, and U represents MGMT promoter unmethylation.

S2

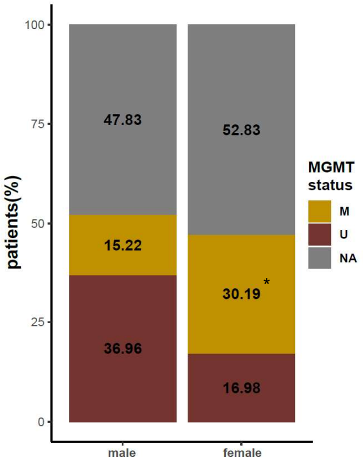

Figure S2. MGMT status between male and female IDH-wildtype GBM patients. M represents MGMT promoter methylation, and U represents MGMT promoter unmethylation.

S3

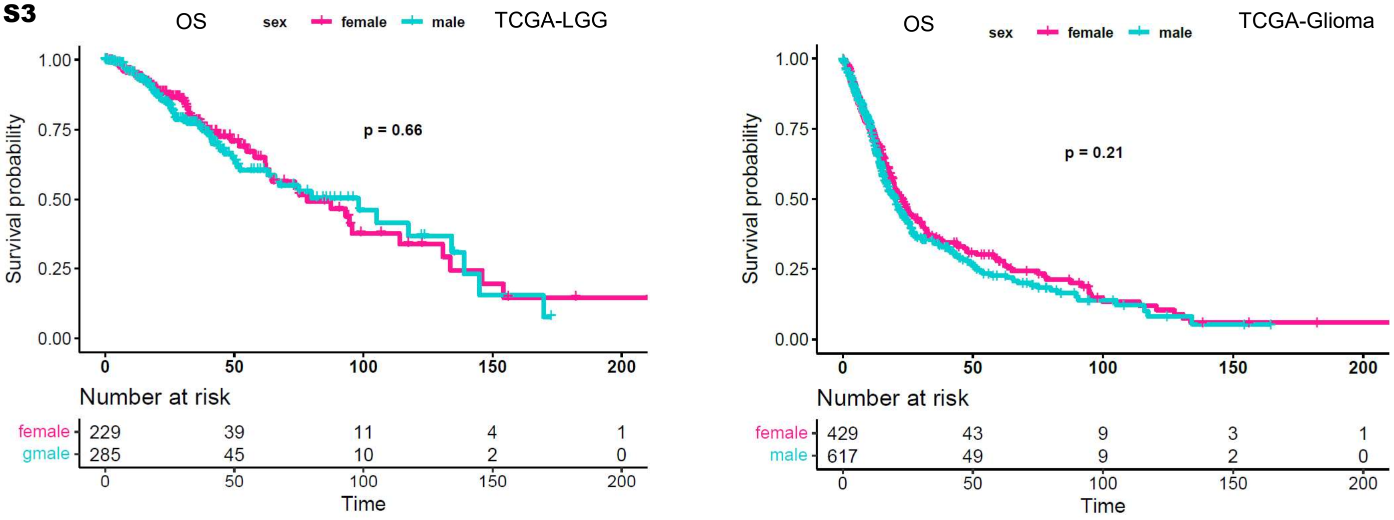

Figure S3. Kaplan−Meier analysis of overall survival in a dataset of LGG and glioma patients from TCGA.

S2

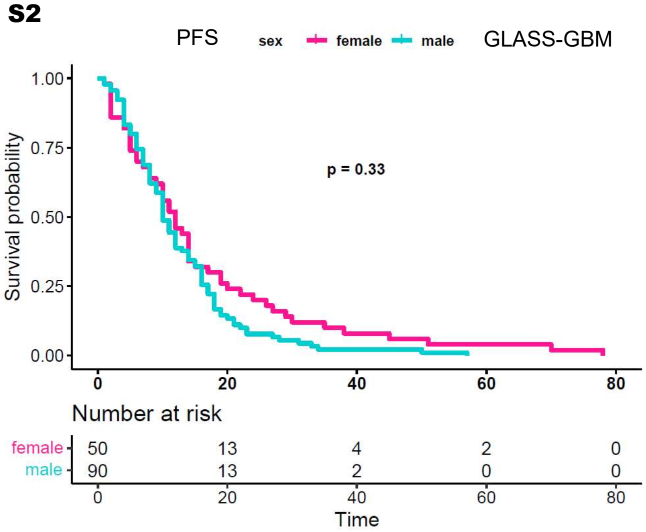

Figure S4. Kaplan−Meier analysis of progression-free survival in a dataset of male and female patients from GLASS.
